# Cost analysis and cost effectiveness of a subsidized community supported agriculture intervention for low-income families

**DOI:** 10.1186/s12966-023-01481-7

**Published:** 2023-07-10

**Authors:** Jennifer A. Garner, Karla L. Hanson, Stephanie B. Jilcott Pitts, Jane Kolodinsky, Marilyn H. Sitaker, Alice S. Ammerman, Donald Kenkel, Rebecca A. Seguin-Fowler

**Affiliations:** 1 School of Health & Rehabilitation Sciences, College of Medicine, The Ohio State University, Columbus, OH USA; 2 John Glenn College of Public Affairs, The Ohio State University, Columbus, OH USA; 3 Master of Public Health Program, Cornell University, Ithaca, NY USA; 4 Brody School of Medicine, Department of Public Health, East Carolina University, Greenville, NC USA; 5 Department of Community Development and Applied Economics, University of Vermont, Burlington, VT USA; 6 Ecological Agriculture and Food Systems, The Evergreen State College, Olympia, WA USA; 7 Gillings School of Global Public Health, School of Medicine, University of North Carolina at Chapel Hill, Chapel Hill, NC USA; 8 Cornell Brooks School of Public Policy, Cornell University, Ithaca, NY USA; 9 Department of Nutrition & Food Science, Texas A&M University, College Station, TX USA

**Keywords:** Community supported agriculture, Cost-offset, Subsidized, Low income populations, Food security, Skin carotenoids, Fruit and vegetable eating behaviors

## Abstract

**Background:**

The food system has a dynamic influence on disparities in food security and diet-related chronic disease. Community supported agriculture (CSA) programs, in which households receive weekly shares of produce from a local farmer during the growing season, have been examined as a possible food systems-based approach for improving diet and health outcomes. The purpose of this study was to estimate the cost of implementing and participating in a multi-component subsidized community supported agriculture intervention and calculate cost-effectiveness based on diet and food security impacts.

**Methods:**

Using data from the Farm Fresh Foods for Healthy Kids (F3HK) randomized controlled trial in New York, North Carolina, Vermont, and Washington (n = 305; 2016–2018), we estimated programmatic and participant costs and calculated incremental cost-effectiveness ratios (ICERs) for caregivers’ daily fruit and vegetable (FV) intake, skin carotenoids, and household food security from program and societal perspectives.

**Results:**

F3HK cost $2,439 per household annually ($1,884 in implementation-related expenses and $555 in participant-incurred costs). ICERs ranged from $1,507 to $2,439 per cup increase in caregiver’s FV intake (depending on perspective, setting, and inclusion of juice); from $502 to $739 per one thousand unit increase in skin carotenoid score; and from $2,271 to $3,137 per household shifted out of food insecurity.

**Conclusions:**

Given the known public health, healthcare, and economic consequences of insufficient FV intake and living in a food insecure household, the costs incurred to support these positive shifts in individual- and household-level outcomes via a F3HK-like intervention may be deemed by stakeholders as a reasonable investment. This work helps to advance a critical body of literature on the cost-effectiveness of subsidized CSAs and other economic and food system interventions for the sake of evidence-based allocation of public health resources.

**Trial registration:**

: ClinicalTrials.gov. NCT02770196. Registered 5 April 2016. Retrospectively registered. https://www.clinicaltrials.gov/ct2/show/NCT02770196.

## Background

The food system has a dynamic influence on disparities in food security and diet-related chronic disease [1]. Community supported agriculture (CSA) programs, in which households receive weekly shares of produce from a local farmer during the growing season, have been examined as a possible food systems-based approach for improving diet and health outcomes [2–4]. Given known disparities in who is reached by CSAs [3] and the potential to exacerbate disparities if interventions are not targeted appropriately [1], cost-offset CSA (CO-CSA) has emerged as a model for expanding access to low-income households via reduce-priced produce shares [5]. CO-CSAs have demonstrated potential to improve fruit and vegetable (FV) intake and other diet- and health-related outcomes [6–8].

Farm Fresh Foods for Healthy Kids (F3HK) was an economic CO-CSA intervention executed in multiple communities across New York, Vermont, North Carolina, and Washington for low-income families with children that incorporated environmental and behavioral supports via the provision of cooking equipment and a series of CSA-tailored, skill-building nutrition and cooking classes [9–11]. Measuring the impacts of complex public health interventions like F3HK has comprised the backbone of public health scholarship, yet evidence of effectiveness alone is insufficient to inform uptake, sustainment, and scaling [12]. The diversity of programmatic options for any given target (e.g., improving FV intake) necessitates that relative costs, alone and in relation to outcomes, be considered toward the strategic allocation of resources by organizational decision makers [13]. The Panel on Cost-Effectiveness in Health and Medicine recommends that measured costs should reflect the opportunity costs of resources used, that is, the marginal value forgone [14]. Moreover, the opportunity costs incurred by participants—that is, the value of time they could have spent engaged in other activities—is an important, yet understudied, consideration. This is especially true given the equity implications of interventions that transfer additional burden onto resource-constrained segments of the population [15, 16].

The objectives of this study were to quantify the costs associated with implementing and participating in the F3HK intervention, and to calculate cost-effectiveness ratios for outcomes demonstrating statistical improvements from baseline to endpoint: caregivers’ FV intake and skin carotenoids, and household food security status [11].

## Methods

The research reported here was completed as part of a multi-state, two-arm randomized controlled trial of F3HK. To be eligible, families had to be willing to enroll both a caregiver over 18 years of age and a child between the ages of 2 and 12 years; be English-speaking; have an annual household income ≤ 185% of the Federal Poverty Level; be either new to CSA or have not participated in one for at least three years; agree to pay for a half-priced CSA share (with weekly Supplemental Nutrition Assistance Program [SNAP] payment structure available); provide an active email address and be able to access the internet for purposes of data collection; and express interest in attending the associated CSA-tailored healthy eating classes. Additional details regarding participant recruitment, randomization, the intervention, and data collection are reported elsewhere [10, 11]. This analysis draws upon caregiver and household outcome data (n = 305) and program and participant cost assessments [11]. All methods and data collection tools were reviewed and approved by the Institutional Review Boards (IRBs) at the University of Vermont (protocol ID: CHRBSS 16–393) and Cornell University (protocol ID: 1501005266).

### Participant outcome measures

Participant outcomes studied in this analysis included caregivers’ self-reported FV intake, caregivers’ skin carotenoids, and household food security status at baseline (in spring) and CSA season end (in fall). Outcomes were assessed using validated tools. Daily FV intake of caregivers in cups was assessed using the National Cancer Institute’s (NCI) All-Day Fruit and Vegetable Screener (FVS) [17]. The FVS was adapted to an online format and images of portion sizes were included to enhance accuracy of estimation [18]. Cups of FV intake per day were calculated with and without juice. Skin carotenoids—an objective biomarker used increasingly as a proxy for FV intake given the carotenoid content of a wide variety of FV—were measured with resonance Raman spectroscopy (RRS) using the Pharmanex© Biophotonic Scanner S3 (NuSkin Enterprises, Provo, UT, USA), and are reported in thousands. These scans were conducted in-person, by trained research staff. RSS technology has been validated in adults [19–22]. RRS scores range from 0 to 89,000, with higher scores reflecting greater skin carotenoid content. Household food security status was measured using the 6-item short form of the U.S. Department of Agriculture Food Security Survey Module (FSSM) with a 30-day reference period [23]. Affirmation of fewer than two survey items resulted in a classification of ‘food secure’.

### Program expense estimations

Expenses associated with F3HK implementation were estimated using program administrative records. The expenses limited to this ‘narrow program perspective’ reflect the burden of resources that programmers may expect to bear: salaries, wages, and benefits; facilities and utilities; and equipment, supplies, and travel. Across all cost categories, care was taken to omit costs related to the conduct of research (e.g., staff time allocated for data collection activities).

Facilities and utilities estimates were approached in two ways. First, they were estimated in accordance with the rate used by universities, like those that collaborated to implement F3HK, to generate “indirect costs” that pay for facilities, utilities, and other overhead expenses. Universities use such funds to cover not only facilities and utilities, but also other resources across campus (e.g., libraries and statistical support) that are not relevant to F3HK implementation; thus, implementation in other contexts would likely cost considerably less. We attempted to simulate a more realistic cost estimate by re-analyzing all ICERs using a facility and utility rate more in-line with non-university settings. Based on correspondence with community partners and websites reporting non-profit indirect rates ranging from 10 to 35%, we opted to use a potentially average rate of 22%.

### Participant cost estimations

To tally expenses from a ‘broad societal perspective,’ the direct expenses and opportunity costs bore by participants taking part in the intervention were added to the costs from the narrow program perspective (i.e., the program implementation costs). Direct participant expenses included half the price of the CSA share and travel-related costs incurred for CSA pick-up and healthy eating class attendance. At endpoint, participants were also asked whether they incurred any routine childcare expenses related to study participation, though children were welcomed to join CSA pick-up and the educational sessions.

Participant expenses related to the CO-CSA share were estimated based on the average value of a 50%-subsidized share across all sites ($13/week) [24], multiplied by the median portion of weeks participants picked up their share (84–88% of weeks, per CSA pick-up logs) [24] and incurred this expense.

Participant expenses related to travel to and from CSA pick-up sites and healthy eating classes were estimated using self-reported data collected at season endpoint. Participants reported the mode by which they traveled to intervention activities; miles traveled; other associated costs (e.g., taxi or bus fares); time in transit (regardless of travel mode); and time typically spent at the pick-up site and, if applicable, the healthy eating classes. Among those who drove a vehicle to intervention activities, the roundtrip mileage was multiplied by the 2017 government mileage reimbursement rate ($0.535). For both CSA pick-up and class attendance, those who walked or took “other” forms of transit were assumed to have zero direct travel expenses. For CSA pick-up, travel expenses per trip were multiplied by the median portion of weeks participants picked up their share per season (18 weeks) and weighted according to the percentage of household using each mode of transit. For class attendance, travel expenses per trip were multiplied by the average number of classes attended (n = 3) based on the F3HK process evaluation [23] and weighted according to the percentage of households using each mode of transit.

Participant opportunity costs included time spent in intervention-related activities (i.e., in transit to and from and at pick-up sites and healthy eating classes) and were also estimated based on self-reported data collected at endpoint. While classes were held outside of traditional work hours, the low-income caregivers engaged in the intervention may have sought to use this time in other valued ways. Reported time spent in relevant activities was summed. Time was valued according to the average applicable state-level minimum wage rates for 2016 and 2017 and weighted by the number of study participants in each state. During the study period, state minimum wage rates averaged $7.25 in North Carolina (applicable for n = 37 intervention participants), $9.35 in New York (n = 45), $9.80 in Vermont (n = 34), and $10.24 in Washington (n = 32), resulting in a weighted hourly rate of $9.12. Median hours spent per participant in intervention-related activities over the course of a program season were multiplied by this rate to estimate the opportunity cost of intervention participation.

### Analyses

F3HK intervention impacts on primary outcomes were estimated using an intent-to-treat framework with multiple imputation of missing data, as reported elsewhere [11]. Outcomes with statistically significant net improvements between baseline and endpoint after adjustment for baseline value—caregiver FV intake, caregiver skin carotenoids, and household food security—were chosen for inclusion in this cost-effectiveness analysis. All net improvements are presented here as unit changes over time from multiple linear regression models. Incremental cost-effectiveness ratios (ICERs) are a metric used to compare the relative resource burden and effectiveness of multiple interventions designed to improve a given outcome [14]. There is no standard of care or programmatic alternatives to which F3HK was compared, so the assumed costs and effectiveness of the theoretical secondary option were set at zero. ICERs were calculated by dividing the cost of the program, per person, by the net change observed for each included outcome measure. An ICER from the narrow program perspective and the broad societal perspective was calculated for each outcome.

## Results

A total of 148 caregiver-child dyads were enrolled in F3HK and randomized to the intervention. Demographic data were reported previously [11]. Most participating caregivers were female (97%), white (76%), and non-Hispanic (94%). Mean caregiver age was 36 ± 7.5 years. Over half (51%) had a college degree or held an advanced degree. Most participating households were composed of two adults (59%) and the most common number of children in participating households was two (36%).

Significant net effects of the F3HK intervention on health-related outcomes are presented in Table 1, including positive effects on caregiver’s self-reported FV intake with and without juice (+ 1.1, *p* = 0.02 and + 1.0 cups, *p* = 0.03, respectively); caregiver’s skin carotenoids (+ 3.3 RRS units in thousands, *p* = 0.01); and household food security (+ 17.7% point increase in the portion of households categorized as food secure, *p* = 0.04). For a sample composed of 305 households, this net change in household food security represents 54 households (i.e., 305*0.177) shifting from food insecure to food secure.

**Table 1:** F3HK Significant Net Effects on Nutrition Outcomes in Four U.S. States, 2016–2017, (n = 305)

Outcomes	Net effect size	*p*
Caregiver’s FV Intake, cups	+ 1.1	**0.02**
Caregiver’s FV Intake w/o juice, cups	+ 1.0	**0.03**
Caregiver’s Skin Carotenoid, RRS score (thousands)	+ 3.3	**0.01**
Household Food Secure, % points	+ 17.7	**0.04**

Significance of intervention status on change in outcome from baseline was tested using multiple linear regression. All p-values presented are from models adjusted for the baseline value of the dependent variable.

### Program expenses

Cost analyses are provided in Table 2. Costs were captured in 2016 & 2017 USD and averaged. The total annual program expenses associated with salaries, wages and benefits was $65,478, including salary and fringe paid to educators (n = 9 in 2016, n = 10 in 2017) at 0.2 FTE (full time equivalents) each, plus half the salary and fringe paid for a site coordinator in each state. Only half of each site coordinator’s salary and fringe were included to account for the portion of their responsibilities that was research-specific and thus not relevant to this analysis. The average indirect rate charged by the universities associated with this study was 48%; accordingly, annual facility and utility costs levied by these universities totaled $31,161. If employing an indirect rate of 22%, annual facility and utility costs would be $14,405. Equipment, supply, and travel costs were estimated to total $42,764. This included half the price of each participant’s CSA share ($20,202; $13/week on average across sites for a mean season length of 21 weeks, multiplied by 74 households per season); costs associated with purchasing the kitchen equipment selected by each intervention household ($7,380); a one-time expense associated with stocking sites with necessary kitchen equipment ($950); small kitchen utensils to use as class attendance incentives ($1,010); F3HK cookbook printing ($264); cooking ingredients for all sites ($3,438); and implementation-related travel expenses incurred by educators and site coordinators ($9,520). Overall program expenses were estimated at $139,403 annually, or $1,884 per household. Using the non-university indirect cost estimate, overall program expenses were estimated at $122,647 annually, or $1,657 per household.

### Participant costs

Participant costs per season totaled $555 per household, inclusive of expenses associated with the subsidized CSA share, travel to and from CSA pick-up sites and healthy eating classes, and the opportunity cost of time spent in intervention-related activities. When added to the per household program expenses for the sake of estimating costs from the ‘broad societal perspective’, per household costs totaled $2,439 per program season.

Participant expenses for the subsidized CSA share averaged $235 per season ($13 per week multiplied by 18 weeks, given a mean season length of 21 weeks and a median of 84–88% of weeks with confirmed pick-up and payment). Full engagement (i.e., payment for all possible shares per season) would have totaled $273, on average, per household per season.

Participant travel expenses averaged $170 per season, including $145 in CSA-related travel and $25 in class-related travel. Households either drove (93.3%), took a taxi (0.8%), walked (3.4%), or took another unknown form of transport to pick up their CSA share. For those who drove, the CSA pick-up site was a median distance of 16 (IQR: 8, 30) miles roundtrip, equating to $145 in per season travel expenses. One household that took a taxi service reported it cost them $40 per month to do so (i.e., $194 per season). Households that reported attending at least one healthy eating class either drove (95%), took the bus (1.2%), walked (2.4%) or took another, unknown form of transportation (1.2%) to get there and back. Those who drove traveled a median of 16 (IQR: 6, 30) miles roundtrip, equating to $26 in per season travel expenses. One household that took a bus paid $2.50 per trip to do so. Full engagement would have totaled $180 and $77 (i.e., $257 total) for travel expenses incurred to pick up all designated CSA shares and attend all healthy eating classes, respectively.

Other direct expenses were incurred infrequently by participants and not factored into average participant costs for the intervention. Namely, seven households reported paying between $0.25 and $0.65 for parking when picking up their CSA share; one household reported paying $0.50 for parking while attending healthy eating classes; and one household also reported paying $10 per class for childcare expenses.

The opportunity cost of participants’ time was estimated at $150 per season. Median time spent traveling to and from CSA pick-up was 30 min and median time spent at the pick-up site was 10 min for a total time cost per trip of 40 min. Given the portion of weeks per season for which participants picked up their share, this equated to 12 h or $109 in opportunity costs. Median time spent in travel round-trip for healthy eating classes was 30 min and median time spent in class was 60 min for a class-related time cost of 90 min per trip. For the average participant who attended three of the nine offered classes, this equated to 4.5 h or $41 in opportunity costs. Full engagement would have totaled $128 in opportunity costs per season for time spent picking up weekly CSA shares and $123 in opportunity costs per season for time spent at healthy eating classes (total: $251).

**Table 2:** Cost Analysis of F3HK Intervention in Four U.S. States, 2016–2017

Cost Categories	University Estimate	Non-University Estimate ^a^
Program Expenses ^b^		
Salaries, Wages, and Benefits	$65,478	$65,478
Facilities and Utilities	$31,161	$14,405
Equipment, Supplies, and Travel	$42,764	$42,764
Program Expenses – Total, Annually	$139,403	$122,647
Program Expenses – Per Household ^c^	$1,884	$1,657
Participant Costs		
Subsidized CSA Share	$235	$235
Participant Travel	$170	$170
Opportunity Costs ^d^	$150	$150
Participant Costs – Per Household	$555	$555
Total Costs – Per Household	$2,439	$2,212

^a^ Universities, such as those associated with this intervention, pay for facilities and utilities by charging for “indirect costs” at a rate likely to exceed that required by industry or nonprofit settings. We conducted a second analysis using a potentially average non-university indirect rate of 22%, which resulted in a lower estimate for ‘facilities & utilities’ and thus for total program expenses.

^b^ Subtotals represent average costs from implementation in 2016 and 2017 (i.e., they reflect an average of 2016 & 2017 USD).

^c^ An average of 74 households were reached by F3HK per year.

^d^ Median time spent in F3HK-related activities per season was 17.5 h. This was multiplied by the average state-level minimum wage, weighted by the portion of participants from each state, resulting in an hourly rate of $9.12.

Cost-effectiveness analyses from both the narrow program and broad societal perspectives were completed for the four health-related outcomes that improved significantly (Table 3). The incremental cost-effectiveness ratios indicate the estimated cost of a one-unit improvement in each outcome. Ratios varied widely across outcomes and perspectives; improving caregivers’ FV intake (excluding juice) ranged from $1,657 to $2,439 per cup per participant, while improving food security status ranged from $2,271 to $3,137 per household.

**Table 3:** Cost-Effectiveness of Improving Nutrition Outcomes via F3HK in University and Non-University Settings^a^, 2016–2017

	Narrow ^b^ (Univ.)	Societal ^c^ (Univ.)	Narrow (Non-Un.)	Societal (Non-Un.)
Total Cost, Per Participant	$1,884	$2,439	$1,657	$2,212
Caregiver’s FV Intake, cups				
Net Effect	+ 1.1	+ 1.1	+ 1.1	+ 1.1
C/E	$1,713	$2,217	$1,507	$2,011
Caregiver’s FV Intake w/o juice, cups				
Net Effect	+ 1.0	+ 1.0	+ 1.0	+ 1.0
C/E	$1,884	$2,439	$1,657	$2,212
Caregiver’s Skin Carotenoid, RRS score (thousands)				
Net Effect	+ 3.3	+ 3.3	+ 3.3	+ 3.3
C/E	$571	$739	$502	$670
Household Food Secure, # of households				
Total Cost ^d^	$139,403	$169,373	$122,647	$152,617
Net Effect	+ 54	+ 54	+ 54	+ 54
C/E	$2,582	$3,137	$2,271	$2,826

^a^ Universities, such as those associated with this intervention, pay for facilities and utilities by charging for “indirect costs” at a rate likely to exceed that required by industry or nonprofit settings. We conducted a second analysis using a non-university indirect rate of 22%.

^b^ Includes salaries, wages, and benefits; facilities and utilities; and equipment, supplies, and travel.

^c^ Includes all costs included in the narrow program perspective as well as those costs—both actual expenses and opportunity costs—incurred by participants taking part in the intervention.

^d^ Food security was calculated as a sample-level outcome, so the numerator used was the total annual cost for university and non-university settings rather than per household cost. For participant-incurred costs, we multiplied the per participant estimate of $555 by the net number of household shifted out of food insecurity, n = 54 (305*0.177), for a total of $29,970.

## Discussion

The estimated costs incurred by F3HK-participating households exceeded $500 per season, representing about one-fifth of total costs. The high administration costs were driven primarily by the team of educators and coordinators required to implement this complex intervention. This investment, while intensive, resulted in multiple positive outcomes: from baseline to endpoint, caregiver’s daily FV intake increased by 1 cup or more; caregiver’s skin carotenoid score increased by over 10% (from an average baseline score of 29,518); and the portion of households categorized as food secure increased to 60% (from the baseline percentage of 43%) [11].

In practical terms, and if we assume a continuous rate of return on such an investment, our analysis suggests that investing $1000 per household in an intervention of this nature could improve caregiver’s FV intake by one-half to two-thirds cup per day if including juice (range across perspectives assumed: 0.45–0.66) and by two-fifths to three-fifths per day with juice excluded (range: 0.41–0.60 across perspectives) over one program season. Whether the magnitude of the trial-observed shifts would be observed across a broader, more generalizable sample of low-income households is unclear, though; we know that F3HK attracted a unique subset of low-income households [24] and that the cost of a subsidized CSA share is still beyond the purchasing power of many families.

Programmatic stakeholders would have a few caveats to consider if looking to implement such an intervention. First, results indicate that, if implemented in a non-university setting, F3HK cost-effectiveness ratios could be 9–12% lower, depending on the perspective assumed. Second, given participants’ poor engagement with the in-person healthy eating classes [24] and that attendance was not associated with improved outcomes [11], that intervention element may not be as worthy an investment or ripe for virtual adaption. The bulk of estimated costs borne by participants were related to CSA purchase and pick-up, so their burden would remain similar without an educational component. Even so, ensuring more convenient CSA pick-up locations for participants may be warranted. A factorial trial design would be necessary to discern the distinct costs and effectiveness of each intervention element and inform optimization of this multi-modal intervention [25].

Cost-effectiveness is a relative metric; its value lies in its use to compare multiple alternative approaches. Unfortunately, the extant literature contains a dearth of analyses to which our results can be compared. Basu et al. (2020) used a microsimulation model to estimate the cost-effectiveness of a subsidized CSA; they concluded that both the CO-CSA and its comparator, an unconditional cash transfer, could generate a sizable cost savings to society [26]. Similarly, Choi et al. (2018) found that a national FV subsidy for Supplemental Nutrition Assistance Program participants could generate societal cost savings via long-term reductions in diet-related chronic disease [27]. The referenced studies make robust contributions to the literature by extrapolating results of randomized trials to the long-term accumulation of disability- or quality-adjusted life years. Our focus on practice-relevant natural units of FV intake enhances the reliability of our findings but precludes direct comparison of results with these studies. In the primary outcome paper on which the Basu et al. (2020) analysis was based, Berkowitz et al. (2019) observed significant improvements in FV Healthy Eating Index sub-scores akin to our observed improvements in FV intake, suggesting that a micro-simulation analysis of our data may have produced similarly positive results [28].

F3HK appears to be more expensive than a farmers market promotion intervention; the estimated expense of $1,884 per household is nearly ten times that incurred to promote farmers market patronage among low-income consumers ($194/household) [29], though it’s unclear if the market value of produce purchased was accounted for in the farmers market intervention as it was for the CO-CSA. Both studies measured skin carotenoid score as a primary outcome; for the farmers market intervention, a 1-unit increase in skin carotenoid score cost $8.10, whereas F3HK cost $0.50–0.74 per 1-unit score increase (or $502-$739 per 1000-unit increase). Importantly, each study used different technology to measure skin carotenoids—Noia et al. (2021) used the Veggie Meter, which generates a score ranging from 0 to 800, whereas we used the Pharmanex© Biophotonic Scanner S3, which generates a score ranging from 0–89,000—so the scores, and cost-effectiveness ratios, may not be comparable.

When comparing F3HK to a variety of interventions designed to promote fruit and vegetable consumption, it demonstrated a generally greater net effect; the positive shift of 1.0-1.1 cups (i.e., about 2 servings) per day exceeded the net effect of most other studies by over 1.5 servings per day [30]. Notably, it was also more expensive per participant than all but one study, a worksite-based cafeteria intervention [31]. Total cost per participant, for F3HK and other interventions, would likely shift if implemented at scale. While some costs may remain fixed (e.g., staffing), other costs may appreciate economies of scale. For example, the per participant price of shares may decrease if the farmers were guaranteed a larger and more regular customer base. The potential for achieving economies of scale with this and other public health interventions is an area ripe for further study.

Despite the methodological hurdles of evaluating cost-effectiveness of public health interventions, [32] such analyses are critical to understanding the relative value of various interventions for purposes of program planning and resource allocation. Public health researchers should consider their role in growing this body of literature. This will afford comparisons of interventions targeting common outcomes of interest (e.g., FV intake) as well as broader syntheses to understand the attributes of public health nutrition interventions that achieve greatest cost-effectiveness. Beard et al. (2022), for example, analyzed factors associated with cost-effectiveness for a wide range of behavior change interventions and found that interventions targeting non-vulnerable participants and those focused on training and persuasion (versus environmental restructuring) were associated with greater relative cost-effectiveness [33]. This highlights both the pragmatic value and need for caution when comparing intervention cost-effectiveness, though; a focus on relative costs and effects should be balanced with considerations of health equity [34].

Strengths of this analysis include using data from a multi-state randomized controlled trial, defining effectiveness using measures of common interest to public health nutrition practitioners, and calculating cost-effectiveness from the narrow program and broad societal perspectives [14]. The granularity of our cost estimates and our explicit valuation of participant burden are important contributions to the literature, and a key limitation of prior analyses. Limitations of this research include use of a relatively small sample; lack of an extended time horizon over which impacts and long-term cost-effectiveness are examined; and the inherent weakness of cost-effectiveness methodology that allows for analysis of only one outcome at a time, which necessarily underestimates the full utility of the estimated investment. Moreover, in an effort to generate results with clear interpretability for practitioners and avoid the limitations of a quality-adjusted life year-focused analyses, [35] we focused judiciously on outcomes of public health relevance and did not estimate shifts in quality- or disability-adjusted life years. Finally, we acknowledge that the intervention did not have an impact on the intervention trial’s primary outcomes, namely the FV intake of children, suggesting it could be a good investment for shifting outcomes at the caregiver and household level, but not at the child level [11].

## Conclusions

In this cost-effectiveness analysis of F3HK, the total estimated cost associated with intervention was $2,439 per household annually, including $1,884 in implementation-related expenses and $555 in participation-related costs per household. The ICERs ranged widely across studied outcomes, from just over $502 for a one thousand-unit improvement in skin carotenoid score to $3,137 to shift one household from food insecure to food secure. Given the health and economic consequences of insufficient FV intake—namely heightened chronic disease and mortality risk and billions of dollars in preventable direct and indirect costs [36, 37] — and of living in a food insecure household, [38, 39] the costs incurred to support these positive individual- and household-level outcome shifts may be deemed by stakeholders as a reasonable investment. This work helps to advance a critical body of literature on the cost-effectiveness of CO-CSAs and other economic food security interventions for the sake of evidence-based allocation of public health resources.

## Data Availability

The datasets used and analyzed during the current study are available from the corresponding author on reasonable request.

## List of Abbreviations

CO-CSA: Cost-Offset Community Supported Agriculture
CSA: Community Supported Agriculture
F3HK: Farm Fresh Foods for Healthy Kids
FSSM: Food Security Survey Module
FTE: Full Time Equivalents
FV: Fruit and vegetable
FVS: Fruit and Vegetable Screener
ICER: Incremental Cost-Effectiveness Ratio
IRB: Institutional Review Board
NCI: National Cancer Institute
RRS: Resonance Raman Spectroscopy
